# Opioids and Their Receptors: Present and Emerging Concepts in Opioid Drug Discovery

**DOI:** 10.3390/molecules25235658

**Published:** 2020-12-01

**Authors:** Mariana Spetea, Helmut Schmidhammer

**Affiliations:** Department of Pharmaceutical Chemistry, Institute of Pharmacy and Center for Molecular Biosciences Innsbruck (CMBI), University of Innsbruck, 6020 Innsbruck, Austria; helmut.schmidhammer@uibk.ac.at

The interest in opioids such as morphine, the prototypical opioid ligand, has been maintained throughout the years. Identification of endogenous opioid peptides and their receptors (µ, mu (MOR); δ, delta (DOR); κ, kappa (KOR); nociceptin (NOP)), along with molecular cloning and elucidation of crystal structures of opioid receptors represent key milestones in opioid research. With its ubiquitous distribution in the central and peripheral nervous systems (CNS and PNS), the opioid system has a central role in modulating pain and other physiological functions and pharmacological responses, with therapeutic as well as unwanted side effects. The dramatic increase in medical use and misuse of opioids with the rising number of opioid-related overdose deaths and diagnoses of opioid-use disorder has led to the 21st century opioid crisis.

The Special Issue, “Opioids and Their Receptors: Present and Emerging Concepts in Opioid Drug Discovery”, includes 11 research articles, one communication and six reviews, with authors from 12 different countries, giving insight into ongoing subjects that span the opioid research field. This issue presents recent advances in medicinal chemistry and pharmacology of new ligands targeting the opioid receptors. Moreover, it highlights current concepts in opioid drug discovery together with strategies to mitigate the deleterious effects of opioids. Central topics of this Special Issue include drug design, structure–activity relationships (SAR), biochemistry of the receptors, understanding of ligand specific actions and the link between therapeutic effects, side effects and molecular mode of action.

The review by Sobczak and Goryński [1] addresses the present opioid epidemic with literature showing that over-the-counter (OTC) opioids are misused as an alternative for illicit narcotics or prescription-only opioids. Three OTC opioid drugs, codeine, dihydrocodeine and loperamide, are discussed, including pharmacology, interactions, safety profiles and how pharmacology is being manipulated to misuse, focusing on abuse prevention and prevalence rates. Relatively easy access to OTC opioids is alarming and requires further attention and discussion on the rescheduling of their availability.

The imperative need for safer therapies for pain and other human disease states involving the opioid system continues to drive the search for novel lead molecules and the development of new mechanism-based treatment strategies. Since the structural elucidation of morphine, its skeleton and its conversion to new analogues has been constantly in the attention of medicinal chemists, aiming to discover therapeutically useful drugs and research tools. Three reports present new research in the field of opioid morphinans [2,3,4].

In their study, Wang et al. [2] elaborated on extended SARs in (−)-*N*-phenethyl analogs of *N*-nor-hydromorphone. Within the designed series, *N*-*p*-chloro-phenethylnorhydromorphone was a bifunctional MOR-DOR ligand with a potent partial agonism at the MOR and a full potent agonism at the DOR. This favorable combination of MOR and DOR activities in vitro was translated in vivo by potent antinociception without respiratory depression in squirrel monkeys after subcutaneous administration. In their communication, Kutsumura et al. [3] reported on SARs between the thiol group-trapping ability of morphinans with a Michael acceptor and anti-*Plasmodium falciparum* activities. Using the DOR antagonist 7-benzylidenenaltrexone (BNTX) as a lead structure, new derivatives were designed and a correlation between the antimalarial activity and the chemical reactivity of the BNTX derivatives with 1-propanethiol was established. Using naltrindole (NTI), the indolo-morphinan DOR antagonist, as a lead, Iwamatsu et al. [4] designed sulfonamide-type NTI derivatives by targeting the effect of the N-substituent on functional activities at the DOR. They revealed SARs among the ligands with activities at the DOR ranging from full inverse agonists to full agonists, with cyclopropyl-sulfonamide (SYK-83) as the most potent full inverse agonist. The new NTI derivatives are expected to be useful tools for investigating interactions of ligands with the DOR, conformational changes of the DOR and induced functional activities.

Endogenous and naturally occurring opioid peptides have continuously served as important leads for the design of peptide analogues, with a repertoire of structural modifications that can be targeted when exploring SARs or focusing on the improvement of their pharmacodynamics and the pharmacokinetics of peptide active compounds. Four research articles [5,6,7,8] focused on this subject.

In their report, Tymecka et al. [5] performed a β^2^-*Homo*-amino acid (β^2^hAA) scan of the selective MOR peptide agonist TAPP (H-Tyr-D-Ala-Phe-Phe-NH_2_) sequence, an analogue of endomorphin-2 and enkephalin-derived DAMGO. Derivatives with (R)- or (S)-β^2^hPhe^4^ bound the MOR with affinities equal to that of TAPP. Combining design strategies, synthesis, binding assays and molecular modeling, they provided additional understanding of SARs of the TAPP sequence. Using a structure-based docking at the MOR and three-dimensional interaction pattern analysis, Dumitrascuta et al. [6] rationalized the experimental results on binding and activation of the MOR by three synthetic analogues of the naturally occurring dermorphin and effective analgesics, DALDA, [Dmt^1^]DALDA and KGOP01. The Dmt (2′,6′-dimethyl-L-Tyr) moiety of [Dmt^1^]DALDA and KGOP01 represented the driving force for the high potency and agonist activity at the MOR. The findings of Tymecka et al. [5] and Dumitrascuta et al. [6] offer significant structural insights into flexible peptide ligand-MOR interactions that are important for further understanding of MOR function and pharmacology, and the future design of peptide-based analgesics.

Cassell et al. [7] explored SAR trends, at the meta-position of Phe^4^, of the endogenous DOR peptide Leu^5^-enkephalin, demonstrating that substitution at this position variously regulated DOR and MOR affinity and G protein activity, enabled the fine-tuning of β-arrestin2 recruitment to both receptors, and increased the plasma stability of the derived peptides. The resulting peptide analogues should be useful tools for studying the role of DOR in cardiac ischemia and the importance of DOR mediated β-arrestin2 signaling in the peptides cardioprotective effects.

The SAR study by Brice-Tutt et al. [8] studied the influence of the Phe residues’ stereochemistry in the macrocyclic tetrapeptide CJ-15,208 (*cyclo*[Phe-D-Pro-Phe-Trp]), from the fungus *Ctenomyces serratus,* and its analogue [D-Trp]CJ-15,208 (*cyclo*[Phe-D-Pro-Phe-D-Trp]) on opioid activity profiles. Unlike the parent peptides, KOR antagonism was exhibited by only one stereoisomer, while another isomer produced DOR antagonism. They identified the stereoisomer [D-Phe^1,3^]CJ-15,208 as a potent antinociceptive after oral administration lacking respiratory depression and locomotor impairment and without preference or aversion in mice.

Natural product medicines have a long history of use in the treatment and prevention of many human diseases. Zerumbone, a sesquiterpene from the wild ginger plant *Zingiber zerumbet* (L.) Smith, produces allodynia and hyperalgesia in animals. Gopalsamy et al. [9] reported on the involvement of potassium channels and opioid receptors (MOR, DOR and KOR) in zerumbone-induced antinociception in a mouse model of chronic constriction injury neuropathic pain after intraperitoneal administration.

As the ongoing worldwide opioid crisis is the result of the use of centrally-acting opioids for controlling pain, the idea of peripheralization of opioids to minimize the activation of central opioid receptors, and as a consequence the unwanted CNS side effects, has stimulated the development of peripherally selective opioid ligands, discussed in a research article [10] and a review [11].

In their study, Zádor et al. [10] reported on a new analogue of codeine, 14-methoxycodeine-6-*O*-sulfate (14-OMeC6SU), as a potent, peripheral MOR agonist. It was more effective than codeine, and equipotent to morphine in inducing antinociception in acute nociceptive pain, and it produced peripherally-mediated anti-hyperalgesic effects in inflammatory pain after subcutaneous administration in rats. Additionally, 14-OMeC6SU showed an improved in vitro and in vivo activity profile compared to codeine-6-*O*-sulfat. Fürst et al. [11] reviewed the consequence of the activation of peripheral MORs in analgesia and analgesic tolerance, along with approaches that enhanced analgesic efficacy and decreased the development of tolerance to opioids at the peripheral sites. They also addressed the advantages and drawbacks of the activation of peripheral MORs on the sensory neurons and gut (leading to dysbiosis) in the development of central and peripheral analgesic tolerance. The reviewed data suggest that the development of peripheral analgesic tolerance to opioids is largely dependent on the pain entity, animal pain model, and the route of administration, local versus systemic.

With the awareness that narcotic addiction is derived from the MOR, the KOR is emerging as a promising target for developing safer therapeutics without the common side effects associated with classical opioids, such as rewarding effects, respiratory depression and overdose. Schmidhammer et al. [12] reviewed recent chemical developments of SARs on diphenethylamines, a new class of structurally distinct and selective KOR ligands, with diverse profiles ranging from potent and selective agonists to G protein-biased agonists and selective antagonists. The first lead molecules in the series included the selective KOR full agonist HS665 and the partial agonist HS666. The combination of target drug design, synthetical efforts and pharmacology of diphenethylamines has enabled the identification of structural elements that determine distinct activity profiles, with the potential as candidates for future drug development for the treatment of pain and neuropsychiatric diseases.

Increasing evidence on the heteromerization of native opioid receptors in discrete brain neuronal circuits with selective targeting of heteromers as a tool to modulate receptor activity, and multifunctional ligands, which simultaneously activate two or more targets to produce a more desirable drug profile, are emerging concepts for the development of novel therapeutic drugs and strategies, presented in the earlier cited report [2] and in two additional research articles [13,14].

Using double-fluorescent knock-in mice co-expressing functional MOR and DOR, Derouiche et al. [13] demonstrated that co-expression of native MOR and DOR in hippocampal neurons alters the intracellular fate of the MOR in a ligand-selective manner, with MOR-DOR co-internalization induced by the MOR-DOR biased agonist CYM51010, the MOR agonist DAMGO and the DOR agonist deltorphin II, but not the MOR agonists morphine and methadone or the DOR agonist SNC80. Their observations pointed out to MOR-DOR heteromerization as a means to fine-tune MOR signaling and neuronal activity with the potential for developing novel innovative therapeutics.

The study by Wtorek et al. [14] targeted the concept of multifunctional ligands, specifically novel hybrids combining opioid pharmacophores with either substance P (SP) or neurokinin receptor (NK1) antagonist fragments, as a strategy for developing effective and safer medications for pain treatment. They reported on opioid agonist/NK1 antagonist Tyr-[D-Lys-Phe-Phe-Asp]-Asn-D-Trp-Phe-D-Trp-Leu-Nle-NH_2_ and opioid agonist/NK1 agonist Tyr-[D-Lys-Phe-Phe-Asp]-Gln-Phe-Phe-Gly-Leu-Met-NH_2_ peptide hybrids with antinociceptive efficacy without inducing analgesic tolerance or constipation in mice after intraperitoneal administration.

Research approaches to diminish opioid liabilities take advantage of the current concept of functional selectivity, with biased ligands (agonists and antagonists) as innovative opportunities for opioid pain therapy and use management, subjects reviewed in [12,15,16,17] and explored in a research article [18].

In their review, Faouzi et al. [15] presented the design and pharmacological outcomes of biased agonists of all opioid receptor types (MOR, DOR, KOR and NOP), aiming at achieving functional selectivity. They described a large number of structurally diverse biased agonists, with the focus on the understanding of the limitations and advantages both in vitro and in vivo that they can provide. Azevedo Neto et al. [16] discussed the accumulated literature on the potential of biased MOR agonists for the development as safer analgesics. They presented the pharmacology of three G protein-biased MOR agonists, oliceridine (TRV130), very recently approved for pain treatment, and PZM21 and SR-17018, in relationship to that of morphine and fentanyl, and proposed that their improved safety profile could be likely attributable to low efficacy partial agonism rather than G protein-bias.

A review by Sadee et al. [17] addressed the less explored area of biased opioid antagonism, where biased MOR antagonists, such as 6β-naltrexol, could serve as modulators of opioid dependence, for improved pain therapy and opioid use management. They proposed a novel receptor model that can account for diverse pharmacological effects of MOR ligands, including biased antagonists.

Using molecular docking and molecular dynamics (MD) simulations at three crystal structures of the MOR, Podlewska et al. [18] explored the distinct activity profiles at the MOR of morphine (unbiased ligand), PZM21 and SR-17018 (G protein-biased MOR agonists) and fentanyl (β-arrestin2-biased MOR agonist). Several shared and distinct receptor-ligand interaction patterns were identified, and specific amino acids were proposed to be of particular interest when designing new G protein-biased MOR agonists.

The diversity among the topics in this Special Issue in the up-to-date reports is a testimony to the complexity of the opioid system that results from the expression, regulation and functional role of ligands and receptors. Moreover, the array of multidisciplinary research areas illustrates the rapidly developing research and translational activities in the opioid drug discovery.

We wish to thank all the authors for their contribution to this Special Issue. It is beyond any doubt that it will serve as a useful reference while also stimulating continued research in the chemistry and pharmacology of opioids and their receptors, with the prospective for developing improved therapies of human diseases, but also improving health and quality of life in general.

## References

[1] Sobczak Ł., Goryński K. (2020). Pharmacological Aspects of Over-the-Counter Opioid Drugs Misuse. Molecules.

[2] Wang M., Irvin T.C., Herdman C.A., Hanna R.D., Hassan S.A., Lee Y.-S., Kaska S., Crowley R.S., Prisinzano T.E., Withey S.L. (2020). The Intriguing Effects of Substituents in the *N*-Phenethyl Moiety of Norhydromorphone: A Bifunctional Opioid from a Set of “Tail Wags Dog” Experiments. Molecules.

[3] Kutsumura N., Koyama Y., Saitoh T., Yamamoto N., Nagumo Y., Miyata Y., Hokari R., Ishiyama A., Iwatsuki M., Otoguro K. (2020). Structure-Activity Relationship between Thiol Group-Trapping Ability of Morphinan Compounds with a Michael Acceptor and Anti-*Plasmodium falciparum* Activities. Molecules.

[4] Iwamatsu C., Hayakawa D., Kono T., Honjo A., Ishizaki S., Hirayama S., Gouda H., Fujii H. (2020). Effects of *N*-Substituents on the Functional Activities of Naltrindole Derivatives for the δ Opioid Receptor: Synthesis and Evaluation of Sulfonamide Derivatives. Molecules.

[5] Tymecka D., Lipiński P.F.J., Kosson P., Misicka A. (2020). β^2^-*Homo*-Amino Acid Scan of µ-Selective Opioid Tetrapeptide TAPP. Molecules.

[6] Dumitrascuta M., Bermudez M., Ballet S., Wolber G., Spetea M. (2020). Mechanistic Understanding of Peptide Analogues, DALDA, [Dmt^1^]DALDA, and KGOP01, Binding to the Mu Opioid Receptor. Molecules.

[7] Cassell R.J., Sharma K.K., Su H., Cummins B.R., Cui H., Mores K.L., Blaine A.T., Altman R.A., van Rijn R.M. (2019). The Meta-Position of Phe^4^ in Leu-Enkephalin Regulates Potency, Selectivity, Functional Activity, and Signaling Bias at the Delta and Mu Opioid Receptors. Molecules.

[8] Brice-Tutt A.C., Senadheera S.N., Ganno M.L., Eans S.O., Khaliq T., Murray T.F., McLaughlin J.P., Aldrich J.V. (2020). Phenylalanine Stereoisomers of CJ-15,208 and [d-Trp]CJ-15,208 Exhibit Distinctly Different Opioid Activity Profiles. Molecules.

[9] Gopalsamy B., Chia J.S.M., Farouk A.A.O., Sulaiman M.R., Perimal E.K. (2020). Zerumbone-Induced Analgesia Modulated via Potassium Channels and Opioid Receptors in Chronic Constriction Injury-Induced Neuropathic Pain. Molecules.

[10] Zádor F., Mohammadzadeh A., Balogh M., Zádori Z.S., Király K., Barsi S., Galambos A.R., László S.B., Hutka B., Váradi A. (2020). Comparisons of In Vivo and In Vitro Opioid Effects of Newly Synthesized 14-Methoxycodeine-6-*O*-sulfate and Codeine-6-*O*-sulfate. Molecules.

[11] Fürst S., Zádori Z.S., Zádor F., Király K., Balogh M., László S.B., Hutka B., Mohammadzadeh A., Calabrese C., Galambos A.R. (2020). On the Role of Peripheral Sensory and Gut Mu Opioid Receptors: Peripheral Analgesia and Tolerance. Molecules.

[12] Schmidhammer H., Erli F., Guerrieri E., Spetea M. (2020). Development of Diphenethylamines as Selective Kappa Opioid Receptor Ligands and Their Pharmacological Activities. Molecules.

[13] Derouiche L., Pierre F., Doridot S., Ory S., Massotte D. (2020). Heteromerization of Endogenous Mu and Delta Opioid Receptors Induces Ligand-Selective Co-Targeting to Lysosomes. Molecules.

[14] Wtorek K., Adamska-Bartłomiejczyk A., Piekielna-Ciesielska J., Ferrari F., Ruzza C., Kluczyk A., Piasecka-Zelga J., Calo’ G., Janecka A. (2019). Synthesis and Pharmacological Evaluation of Hybrids Targeting Opioid and Neurokinin Receptors. Molecules.

[15] Faouzi A., Varga B.R., Majumdar S. (2020). Biased Opioid Ligands. Molecules.

[16] Azevedo Neto J., Costanzini A., De Giorgio R., Lambert D.G., Ruzza C., Calò G. (2020). Biased versus Partial Agonism in the Search for Safer Opioid Analgesics. Molecules.

[17] Sadee W., Oberdick J., Wang Z. (2020). Biased Opioid Antagonists as Modulators of Opioid Dependence: Opportunities to Improve Pain Therapy and Opioid Use Management. Molecules.

[18] Podlewska S., Bugno R., Kudla L., Bojarski A.J., Przewlocki R. (2020). Molecular Modeling of µ Opioid Receptor Ligands with Various Functional Properties: PZM21, SR-17018, Morphine, and Fentanyl—Simulated Interaction Patterns Confronted with Experimental Data. Molecules.

